# Trends in the treatment of urinary stone disease in Turkey

**DOI:** 10.7717/peerj.5390

**Published:** 2018-07-31

**Authors:** Kadir Yildirim, Mahmut Taha Olcucu, Muhammed Emre Colak

**Affiliations:** 1 Urology, Training and Research Hospital, Elazig, Turkey; 2 Urology, Agri State Hospital, Agri, Turkey; 3 Software Engineering, Firat University, Elazig, Turkey

**Keywords:** Survey, SWL, Trend, Urolithiasis, Endourology, Stone

## Abstract

**Introduction:**

In this study, a survey was prepared for urologists that asked about their primary choice of treatment for urolithiasis in daily practice and their answers were evaluated.

**Methods:**

The survey was prepared on the Google Docs website and it was sent to 1,016 urologists via email with 752 confirmed deliveries. In addition to the demographic questions about each participant’s age, gender, and institution, the survey presented case scenarios focusing on their preferred treatment modalities for distal ureteric, proximal ureteric, and renal calculi. The participating urologists were divided into two groups according to the frequency that they treat urolithiasis patients.

**Results:**

Of the 752 surveys delivered, 211 urologists (28.05%) responded and 204 answered all questions. According to the results, there were no significant differences between the treatment approaches and the other localizations, but there was a statistically significant difference for treatment approaches to lower pole stones between two groups. In response to the question of which stone treatment method was used less frequently, 124 (60.7%) participants answered that they used shock wave lithotripsy less in the last 10 years.

**Conclusion:**

The present study has shown that while the management of renal and ureteric calculi by Turkish urologists is highly varied, the overall treatment patterns are in accordance with the European Association of Urology guidelines. However, similar to the global trend extracorporeal shock wave lithotripsy is less preferred by Turkish urologists.

## Introduction

New technological developments in endourology have resulted in dramatic changes in the management of stone diseases (Canes & Desai, 2008; Dede et al., 2015; Lee & Bariol, 2011; Resorlu et al., 2014). In general, stone diseases have reaped more benefits from technological advances than the other areas of urology. During the last 30 years or so, we have been witnessing the competition between treatment modalities, with new players constantly being added to the race. In the 1970s, percutaneous nephrolithotomy (PNL) was the most popular method used in the endourology practice. However, in the 1980s, it lost its popularity to extracorporeal shock wave lithotripsy (SWL), but regained its popularity in the 1990s. The reason is because since the early 2000s, lasers and flexible instruments have become popular.

Today, we have many alternatives for the treatment of stone disease when determining the best treatment in accordance with the minimal urological treatment philosophy. In spite of the clear benefits of the noninvasive methods for stone removal, like SWL, there seems to be a present trend in favor of endourology (Oberlin et al., 2015; Rassweiler et al., 2013, 2014; Rosa et al., 2013; Schnabel et al., 2015). New technologies, which include the miniaturization of instruments and the improvement of laser lithotripters, have attracted urologists who normally favor surgery. There is a trend for less use of SWL according to the literature, although still recommended by European Association of Urology (EAU) guidelines (Klein et al., 2018; Lorber et al., 2010).

For the present study, we prepared a survey for urologists in which we questioned their preferences for a first-choice stone treatment in daily practice.

## Methods

We surveyed Turkish urologists between February and March of 2016 using an online questionnaire. The questionnaire was prepared on the Google Docs website and sent to 1,016 urologists via email. The email addresses of 264 physicians were inactive, according to the email delivery system, so 752 questionnaires were actually delivered to urologists. The website does not allow the surveyors access to the respondents’ identities. Participants were from university hospitals, training and research hospitals, government hospitals, private hospitals, and urologists have consisted of academic degrees at all levels in Turkey.

The urologists were invited to participate to the survey via email, and two follow-up emails were sent at 2-week intervals. All the conditions and phases of our study were conducted in accordance with the Helsinki Declaration, and Firat University Ethics Committee approved this research (approval number 17-07).

The questionnaire administered to the participants was composed of demographic questions about their age, gender, and institution. In addition, the survey presented case scenarios focusing on the treatment modality favorites for distal ureteric, proximal ureteric, and renal calculi. When preparing the questions, the sizes, and localizations of the stones were determined using the reference values of the EAU guidelines. However, the respondents were asked to select the treatment modality that they used as their first choice for clinical practice, not the EAU recommendations, in the absence of follow-up options. The participants were also divided into two groups according to the frequency with which they treated stone patients. The Group I consisted of those treating stone patients ≤30% of the time, and Group II consisted of those treating stone patients >30% of the time.

After an overall 2-month reply period, the data were entered into the database and analyzed with the Statistical Package for the Social Sciences version 16.0. The statistical analyses were done using the chi-squared test. The data were shown as the percent or standard error of the mean, and the statistical significance was set at *p* < 0.05.

## Results

Of the 752 questionnaires sent, 211 (28.05%) received replies and 204 of these had complete answers. The clinical experience of the participants ranged from 4 to 36 years. The number of participants from university hospitals was 74 (36.2%), while the numbers from training and research hospitals, state hospitals, and private hospitals were 36 (17.6%), 45 (22.05%), and 49 (24.01%), respectively. Table 1 shows the descriptive features of the participants.

**Table 1:** Academic status, experience, and institutions of the participants.

Academic status	Experience (years)	University hospital	Research hospital	State hospital	Private hospital	Total (*n*)
Resident	4.72 + 0.46	11	2	0	0	13
Specialist	12.12 + 7.21	7	27	45	34	113
Assistant professor	11.8 + 5.3	15	1	0	2	18
Associate professor	20.1 + 8.0	23	3	0	6	32
Professor	31.5 + 8.0	18	3	0	7	28
Total	15.2 + 9.9	74	36	45	49	204

The participants were asked “Which stone treatment methods are practically applied in clinically appropriate patients at your department?” In response, 71% answered SWL, 99% answered semirigid ureterorenoscopy (URS), 72% answered flexible URS, 80% answered PNL, and 48% answered laparoscopic stone surgery. In addition, the participants’ first treatment preferences were requested for each of the following cases: lower calyx, kidney, and ureter stones of various sizes. Table 2 shows the responses given to the questions.

**Table 2:** First treatment preferences of the participants according to stone localization.

Stone localization	SWL (%)	PNL (%)	Flex URS (%)	Rigit URS (%)	Laparoscopy (%)	Open (%)
<1 cm lower calyx	49.5	6.4	44.1	n/a	–	–
1–2 cm lower calyx	26.1	38.4	35.1	n/a	–	–
1–2 cm upper, midle, pelvis	61.2	12.1	26.7	n/a	–	–
>2 Kidney stone	2.4	87.3	8.3	n/a	0.5	1.5
>1 cm prox ureter	36.1	–	26.3	31.7	2.9	1
<1 cm prox ureter	57.8	–	16.2	25.5	0.5	–
>1 cm distal ureter	3.4	n/a	1.5	94.1	0.5	0.5
<1 cm distal ureter	5.4	n/a	1.5	93.1	–	–

**Note:**

n/a, Not applicable.

The participants’ responses to the following question ranged between 5% and 75%: “How many of the patients who applied to your clinic are stone patients?” The mean value of the statistical evaluation was 28.82% ± 14.2 (median 30%). The participants were then divided into two groups: Group I (≤30%) was below median value and Group II (>30%) was above this value. When the treatment preferences of lower pole stones smaller than one cm in the first group were considered, the preference rates of SWL, Flexible URS, and PNL were 59.1%, 37.6%, and 3.2%, respectively. In the second group, these ratios were 40.3%, 50.0%, and 9.6%, respectively. There was a statistically significant difference between the treatment approaches in the lower pole stones between the groups (*p* = 0.01), but there were no statistically significant differences between the treatment approaches in the other localizations (*p* = 0.5). Table 3 shows the comparison of the results in terms of the treatment preferences.

**Table 3:** Treatment preferences according to stone localization between groups.

Stone localization	**Group I (*n*)	***Group II (*n*)	*p*
<1 cm lower calix			
SWL	55 (59.1%)	42 (40.3%)	
Flexible URS	35 (37.6%)	52 (50.0%)	0.01*
PNL	3 (3.2%)	10 (9.6%)	
1–2 cm lower calix			
SWL	31 (32.6%)	19 (18.4%)	
Flexible urs	25 (26.3%)	45 (43.7%)	0.01*
PNL	39 (41%)	39 (37.9%)	
1–2 cm upper, middle, pelvis			
SWL	61 (62.9%)	63 (60.6%)	
Flexible urs	23 (23.7%)	31 (29.8%)	0.5
PNL	13 (13.4%)	10 (9.6%)	
>2 Kidney stone			
SWL	3 (3.2%)	2 (1.9%)	
Flexible urs	11 (11.7%)	4 (3.9%)	0.1
PNL	80 (85.1%)	96 (94.1%)	

**Notes:**

* *p* < 0.05.

** Group I: rate of treated stone patients ≤30%.

*** Group II: rate of treated stone patients >30%.

In the results of this study, differences were seen in the treatment approaches among the institutions; for instance, the endourological intervention rates in the private hospitals were higher than those in the other institutions. The SWL rates showed that it was the least favored treatment modality for private hospitals when compared to other hospitals. While this was statistically significant in kidney stones between one and two cm (42.9%) and in proximal ureter stones >1 cm (34.7%) (*p* < 0.05), it was not statistically significant in the other localizations (*p* > 0.05).

Overall, there were no statistically significant differences between the treatment preferences in the analysis results with regard to the participants’ academic degrees (*p* > 0.05).

**Figure 1:**
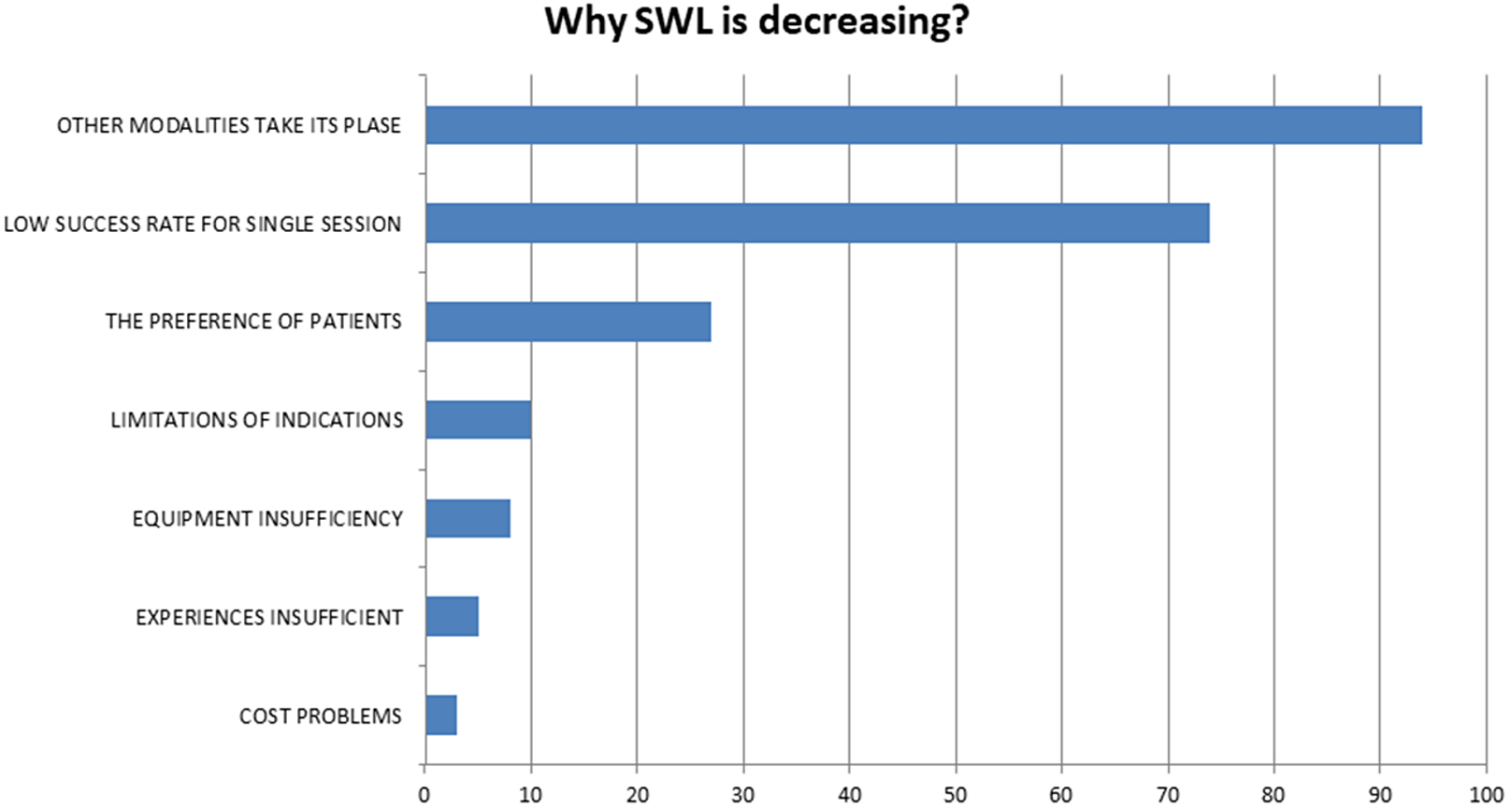
The reasons for decrease in SWL according to participants.

In answer to which of the stone treatment modalities had been used less for the last 10 years, SWL was used less by 124 (60.7%) of the participants, while 58 (28.4%) of the participants answered laparoscopic stone surgery, 21 (10.2%) answered PNL, and 1 (0.5%) answered flexible URS. In addition, 124 of the participants were asked why they believed that SWL usage decreased over time, and their responses are summarized in Fig. 1.

## Discussion

The trend among Turkish urologists seems to be in favor of endourology. According to results of this study SWL less preferred by Turkish urologists parallel to global tendency.

Urolithiasis treatment methods have been rapidly changing and evolving, and advances in imaging techniques have led to smaller stones being seen more often in daily practice. The miniaturization of surgical instruments and the improvements in endoscopic technology have expanded the range of instruments that can be used in stone therapy today (Inoue et al., 2018; Raheem et al., 2017; Resorlu et al., 2013). Despite this, SWL is still considered to be the first-line treatment for renal stones up to one to two cm and most radiopaque ureteral calculi. However, a consistent trend favors endourology (Rassweiler et al., 2014; Schnabel et al., 2015; Soylemez et al., 2013).

The current study participant response rate was 28.05%. Similar to our study Soylemez et al. (2012) reported that 26.8% response rate in their study. Moreover, Dauw et al. (2015) and Ifediora & Rogers (2018) reported that there was a 20.68%, and 11.3% response rate in their surveys, respectively. According to these data, our response rate was consistent with previous studies (Soylemez et al., 2012, 2013).

Treatment decisions of stone diseases depends on stone characteristics, infundibulopelvic angle, infundibular length and width, patients comorbidities, etc., when the case scenarios were determined, the guidelines were used as reference values. The urologists were asked which treatment modality they preferred in daily practice. We questioned them what your first choice would be in a case scenario where follow up is not an option and all treatments can be applied for patients renal or ureteral stones. In the lower pole stones that were smaller than one cm, 44.1% of the participants preferred URS, 49.5% of them preferred SWL and 6.4% preferred PNL, but in one to two cm lower pole stones this ratios changes to 35%, 26.1%, and 40%, respectively. For one to two cm stones in the upper calix and middle pole and pelvic stones, the participants stated that they preferred SWL (61.2%). For kidney stones larger than two cm, PNL was preferred by 87% of the participants. It is surprising that urologists who deal with more stone disease in their current practice prefer flexible URS or PNL to SWL as the first choice in lower pole stones. This may be explained by the desire to reach a higher stone-free rate sooner. In the case of small lower pole stones (one cm), 59% of those in Group I preferred SWL, whereas this ratio was 42% in Group II (*p* < 0.05). In the one to two cm lower calix stones, 32% of Group I preferred SWL, while this ratio was 18% in Group II (*p* < 0.05). According to the results of this study, the claims about SWL losing its popularity are confirmed (Rassweiler et al., 2011, 2014).

If the aim is to present a less invasive treatment option for patients, then why has the noninvasive and efficacious SWL lost its popularity to endourological modalities? For the question “Which treatment options are less popular today?” 60.7% of the respondents gave SWL as an answer. When questioned about the factors leading to this situation, a large proportion of the participants indicated that new treatments are being used instead, and that the success rate in one session is low. As such, 30% of the participants stated that the patients were not in favor of this treatment option.

According to the results of this study, SWL is less preferred by Turkish urologists; however, the literature shows that this situation is not specific to Turkey. In their case series, Rassweiler et al. (2014) reported that there has been a significant increase in URS procedures, but a significant decrease in SWL procedures since 2000. Another important indication of this is the change in the number of publications on the applied treatment in the literature over the years. Tiselius & Chaussy (2015) compared the publication numbers for PNL, URS, and SWL between 1985 and 2013 and noted a dramatic increase in the endourological treatment numbers, although there were no significant changes in the SWL numbers.

Unfortunately, the fascinating developments in endourology have led to the lesser usage of SWL than it deserves. The safety and efficacy of SWL have been proven over more than 30 years of treatment (Fuchs et al., 1985; Lawler et al., 2017; Lingeman et al., 2009; Rassweiler et al., 2001). Moreover, SWL has the shortest hospital stay length and does not require anesthesia (Elmansy & Lingeman, 2016).

We suggest that if SWL, which is a noninvasive method, is applicable in a specific case, the other methods should not be considered primarily because endourological treatments are invasive, even if at a minimal level. For the proper localization and stone size, SWL deserves more consideration.

## Conclusion

The present study has shown that while the management of renal and ureteric calculi by Turkish urologists is highly varied, the overall treatment patterns are in accordance with the EAU guidelines. However, similar to the global trend SWL is less preferred by Turkish urologists.

## Supplemental Information

10.7717/peerj.5390/supp-1 Supplemental Information 1 Raw data.

10.7717/peerj.5390/supp-2 Supplemental Information 2 Question forms.

## Additional Information and Declarations

### Data Availability

The following information was supplied regarding data availability: The raw data were uploaded as a Supplemental File.
