# Supplementary material for: Trends in the treatment of urinary stone disease in Turkey
Source: PeerJ. 2018 Jul 31;6:e5390. doi: 10.7717/peerj.5390 (PMC6074772; doi:10.7717/peerj.5390)
Supplement: Supplemental Information 2 [file peerj-06-5390-s002.docx]

**Questions of surveys**

Q 1) How long have you been working in urology?

Q 2) Which institution do you work in?

- University hospital
- Training and research hospital
- State hospital
- Private hospital

Q 3) What is your academic title?

- Resident in urology
- Specialist
- Assistant professor
- Associate professor
- Professor

Q 4) Among the patients who applied to your clinic, the average rate of stone disease is%? (Any number from 0 to 100)

Q 5) Which of the following endourological stone treatment modalities are actively applied to the appropriate patients in your clinic?

- ESWL
- Semirigit URS
- Flexible URS
- PNL
- Laparoscopic stone surgery

6-13 questions, rather than the EAU guidline information, which treatment modalities you prefer in your current practice is questioned.

Q 6) What is your first treatment option in lower calyx stones smaller than 1 cm?

- ESWL
- PNL
- FLEXIBLE URS

Q 7) What is your first treatment option in lower calyx stones between 1 cm and 2cm?

- ESWL
- PNL
- FLEXIBLE URS

Q 8) What is your first treatment option in upper, midle and pelvis stones between 1cm and 2cm?

- ESWL
- Flexible URS
- PNL
- Laparoscopic stone surgery
- Open surgery

Q 9) What is your first treatment option in kidney stones bigger than 2 cm?

- ESWL
- Flexible URS
- PNL
- Laparoscopic stone surgery
- Open surgery

Q 10 )What is your first treatment option in proximal ureteral stones bigger than 1 cm?

- ESWL
- Antegrad URS
- Semirigit URS
- Flexible URS
- Laparoscopic stone surgery
- Open surgery

Q 11 )What is your first treatment option in proximal ureteral stones smaller than 1 cm?

- ESWL
- Semirigit URS
- Flexible URS
- Laparoscopic stone surgery
- Open surgery

Q 12 ) What is your first treatment option in distal ureteral stones bigger than 1 cm?

- ESWL
- Semirigit URS
- Flexible URS
- Laparoscopic stone surgery
- Open surgery

Q 13 )What is your first treatment option in distal ureteral stones smaller than 1 cm?

- ESWL
- Semirigit URS
- Flexible URS
- Laparoscopic stone surgery
- Open surgery

Q 14 ) 14) which of the following endourological stone treatment options do you think has decreased in the last 10 years?

- ESWL
- PNL
- Flexible URS
- Laparoscopic stone surgery

Q 15) 14. Which of the following can be said as the reason for the answer given in the question?

- Other modalities take it place
- Low success rate for single season
- The preference of patients
- Limitations of indications
- Equipment insufficiensy
- Experiences insufficients
- Cost problems
